# Elective delivery versus expectant management for pre-eclampsia: a meta-analysis of RCTs

**DOI:** 10.1007/s00404-016-4281-9

**Published:** 2017-02-02

**Authors:** Yonghong Wang, Min Hao, Stephanie Sampson, Jun Xia

**Affiliations:** 1 The Second Hospital of Shanxi Medical University, 382 WuYi Road, TaiYuan City, Shanxi Province 030001 China; 2 Systematic Review Solutions. Ltd, Yan Tai, China; 3 Psychiatry Department, The University of Nottingham, Nottingham, UK

**Keywords:** Pre-eclampsia, Expectant management, Deliver, Obstetric, Meta-analysis

## Abstract

**Purpose:**

To evaluate the effectiveness and safety of elective delivery versus expectant management for women with pre-eclampsia (PE) and to assess neonatal outcomes before and after 34 weeks gestation.

**Methods:**

We searched Biomed Central, CINAHL, Cochrane Library, Embase, HMIC, Medline, and WHO trial registry, British Nursing Index, ClinicalTrials.gov, Current Controlled Trials, and Web of Science on 16 March, 2016. 1704 citations were identified. Randomised controlled trials comparing elective delivery with expectant management for women with PE were included. Seven studies were included (*n* = 1501). There were no maternal deaths.

**Results:**

Elective delivery lowered incidence of complications in women with PE or hypertension greater than 34 weeks gestation (*n* = 756; RR, 0.64; 95% CI 0.51–0.80). For women with severe PE less than 34 weeks gestation, elective delivery lowered the incidence of placental abruption (*n* = 483, 5 RCTs; RR, 0.43; 95% CI 0.19–0.98). For women with PE or hypertension greater than 34 weeks gestation, elective delivery also reduced the need for antihypertensive drug therapy. The need for ventilatory support and the risk of developing neonatal intraventricular hemorrhage or hypoxic ischemic encephalopathy may be increased in infants whose mothers undergo elective delivery for severe PE at less than 34 weeks gestation. However, there was no relevant evidence for women with severe PE over 34 weeks.

**Conclusions:**

In women with PE or gestational hypertension beyond 34 weeks gestation, elective delivery can decrease the incidence of complications, severe hypertension and the need for antihypertensive drug therapy. Elective delivery can also lower the risk of placental abruption in women before 34 weeks gestation with severe PE, however, may be associated with increased risk of neonatal complications.

## Introduction

Pre-eclampsia (PE) is a pregnancy-specific condition characterized by hypertension and consequent damage to other organs (e.g. kidney, liver) [1]. PE occurs in approximately 2–8 % of pregnancies, typically in the second or third trimester, and is considered as one of the most common, dangerous, and unpredictable complications of pregnancy [1–3]. Women with PE are at an approximately fourfold higher risk of death than those without PE. Additionally, babies born to mothers with PE have substantially increased odds of death and severe complications [4]. Common risk factors for PE are listed in Table 1 [3, 5]. While the exact mechanisms underlying PE remain unclear, some evidence suggests that it may be related to inadequate blood supply to the placenta and the resultant hypoxic environment [6]. Infants born after PE are at increased risk of “small for gestational age”, and severe and early onset PE were associated with significant fetal growth restriction [7].

**Table 1:** Common risk factors for pre-eclampsia

*Pregnancy-specific issues*
Nulliparity
Partner-related factors [new paternity, limited sperm exposure (e.g. barrier contraception)]
Multifetal gestation
Hydatidiform mole
*Maternal pre-existing conditions*
Older age
African-American race
Higher body mass index
Pregestational diabetes
Chronic hypertension
Renal disease
Antiphospholipid antibody syndrome
Connective tissue disorder (e.g. systemic lupus erythematosus)
Family history or pre-eclampsia
Lack of smoking

According to published clinical guidelines, the management of PE is primarily dependent on two important factors: the gestational age and the severity of the disease [8–10]. Because delivery is the only curative treatment for PE, the timing of delivery is critical for clinical outcomes. The American College of Obstericians and Gynecologists (ACOG) taskforce bulletin indicates delivery at 37 weeks of gestation for women with PE (including gestational hypertension) [10]. Although preterm delivery may be considered for women with severe PE, clinicians should carefully evaluate the serious consequences and adverse outcomes associated with PE progression over the risks of preterm birth. There is controversy regarding the benefits of elective delivery over expectant management before 34 weeks of gestation for women with PE. The National Institute for Health and Clinical Excellence (NICE) guidelines generally recommend not to perform elective delivery for PE management before 34 weeks of gestation unless severe refractory hypertension or above-threshold (pre-documented in a consultant plan) maternal or fetal indications develop after a course of corticosteroids treatment [9]; while the ACOG taskforce bulletin states that “continued pregnancy should be undertaken only at facilities with adequate maternal and neonatal intensive care resources for women with severe PE at less than 34 weeks of gestation” without providing further instructions as to whether elective delivery can be performed if certain conditions occur [10]. Despite these guideline recommendations, many clinicians still consider that active PE intervention after 34 weeks of gestation promotes a better outcome for both the mother and neonate. However, evidence supporting these management criteria is very limited. A recently published Cochrane review compared the effects of a policy of interventionist care and early delivery (before 34 weeks of gestation) with a policy of expectant care and delayed delivery for women with early onset severe PE, but women with non-severe PE were not included in the analysis [11]. In addition, although the Cochrane review reported that expectant management may be associated with decreased infant mortality before 34 weeks of gestation, it was unable to reach a conclusion regarding maternal outcomes because of insufficient data, therefore, more evidence will be required to provide guidance regarding management of PE, in general, and severe PE before 34 weeks’ gestation [11].

The objectives of this meta-analysis were to compare (1) the maternal and fetal outcomes of elective delivery versus expectant management; (2) the optimal timing of delivery (before 34 weeks of gestation versus after 34 weeks of gestation) for preventing PE-associated complications. We classified the patient population as having “PE in general” and “severe PE” and performed separate analyses for each patient group.

## Materials and methods

Sources. The following databases were searched from their establishment dates to 29 June, 2014: Biomed Central; British Nursing Index; CINAHL; Cochrane Library; ClinicalTrials.gov; Current Controlled Trials; Embase; HMIC; Medline; Web of Science; and the WHO trial registry. After the trial search, the review protocol was registered on PROSPERO International Prospective Register of Systematic Review. The registration code is CRD42013004741 [12]. We updated the search at 16 March, 2016.

*Study selection* The severity of PE is determined by both clinical features and the presence of certain laboratory abnormalities. We adopted the diagnostic criteria for PE and severe PE stated in American College of Obstetricians and Gynecologists (ACOG) guideline. The diagnostic criteria were presented it in Table 2 [13, 14]. Severe PE is associated with major adverse outcomes, such as seizures, hemorrhagic and ischemic strokes, placental abruption and stillbirth. “HELLP syndrome” is a variant of severe pre-eclampsia that is characterized by hemolysis, elevated liver enzymes, and low platelets. Hepatic failure, liver rupture, renal dysfunction or irreversible renal failure secondary to renal cortical necrosis has also been reported [15]. As defined in ACOG guideline, the gestational hypertension is ‘BP elevation after 20 weeks of gestation in the absence of proteinuria or the aforementioned systemic findings [13].

**Table 2:** Diagnostic criteria for pre-eclampsia and severe pre-eclampsia [13, 14]

Name	Diagnostic criteria
Pre-eclampsia	Hypertension (a blood pressure greater than or equal to 140 mmHg systolic or equal to 90 mmHg diastolic on two occasions at least 4 h apart after 20 weeks of gestation in a women with a previously normal blood pressure) or severe hypertension (systolic blood pressure ≥160 mmHg or diastolic blood pressure ≥110 mmHg on two occasions 4 h apart) *And* Proteinuria (‘≧300 mg per 24-h urine collection’ or ‘Protein/creatinine ratio ≧0.3 mg/dL’ ‘Dipstick reading of 1+, used only if other quantitative methods not available’) *Or* In the absence of proteinuria, new-onset hypertension with the new onset of any of the following: thrombocytopenia, impaired liver function, the new development of renal insufficiency, pulmonary edema or new onset cerebral or visual disturbance
Severe pre-eclampsia	Severe hypertension alone: systolic blood pressure ≥160 mmHg or diastolic blood pressure ≥110 mmHg Or severe hypertension with the following criteria Severe proteinuria at least 3 g (range 2–5 g) protein in 24 h, or 3+ on dipstick Oliguria <500 cc/day upper abdominal pain, pulmonary oedema Neurological disturbances (such as headache, visual disturbances, and exaggerated tendon reflexes) Impaired liver function tests, high serum creatinine, low platelets Suspected intrauterine growth restriction or reduced liquor volume

We included studies if they were (1) randomised controlled trials, (2) evaluated any methods of elective delivery (induction of labour or caesarean section) versus expectant management (policy of delayed delivery), and (3)included treatment of women with pre-eclampsia (however defined) or gestational hypertension, who either before or at-term delivery (up to and greater than 34 weeks). Cluster-randomised studies and studies with a quasi-random design, such as allocation by alternation, day of week, or hospital numbers were excluded, as they have a greater potential for bias [16]. Studies with a crossover design were also excluded, since such a design is not possible with this intervention.

Outcomes. Our primary maternal outcomes included (1) death, (2) eclampsia, and (3) stroke, and (4) any serious morbidity or complications (defined as at least one complication of stroke, placental abruption, kidney failure, liver failure, HELLP syndrome, disseminated intravascular coagulation, pulmonary edema and postpartum hemorrhage). Maternal secondary outcomes included: severe hypertension and need for hypertensive drug therapy. Primary neonatal outcomes included (1) stillbirth, (2) perinatal death, and (3) neonatal death. Secondary neonatal outcomes included: necrotizing enterocolitis, requirement for ventilatory support, cerebral hemorrhage, interventricular hemorrhage or hypoxic ischemic encephalopathy, hyaline membrane disease, bronchopulmonary dysplasia and pneumothorax and small-for-gestational age.

Data extraction and management. Review authors YW and Min Hao (MH) independently extracted data relating to our outcomes of interest into an electronic proforma. We sub-categorized results into groups according to gestational age at trial entry: less than or greater than 34 weeks of pregnancy. We carried out the statistical analysis using the Review Manager v5.3 software, using a fixed-effect model for meta-analysis combining data where trials examined the same intervention, and the trials’ populations and methods were judged sufficiently similar. For binary/dichotomous outcomes, we obtained estimates of the treatment effect using the risk ratio (RR) and its 95% confidence interval (CI). For continuous outcomes, we used a mean difference (MD). Where we suspected clinical or methodological heterogeneity between studies, sufficient to suggest that treatment effects may differ between trials, we used a random effects model. We investigated heterogeneity between studies by considering the *I* ^2^ method alongside the chi-square *p* value.

Assessment of risk of bias. The Cochrane Handbook for Systemic Reviews of Interventions was considered when assessing risk of bias in the included studies [17]. Methodological quality was rated as either a ‘low’, ‘high’ or ‘unclear’ risk of bias based on domains, including randomization, allocation concealment, blinding, incomplete outcome data and selective reporting.

Assessment of the quality of evidence. The GRADE methodology was applied for evaluating the quality of evidence for the primary maternal outcomes that the authors had consistently identified as important. We judged the level of evidence based on the instruction given in the GRADE [16].

## Results

### Study selection

**Fig. 1:**
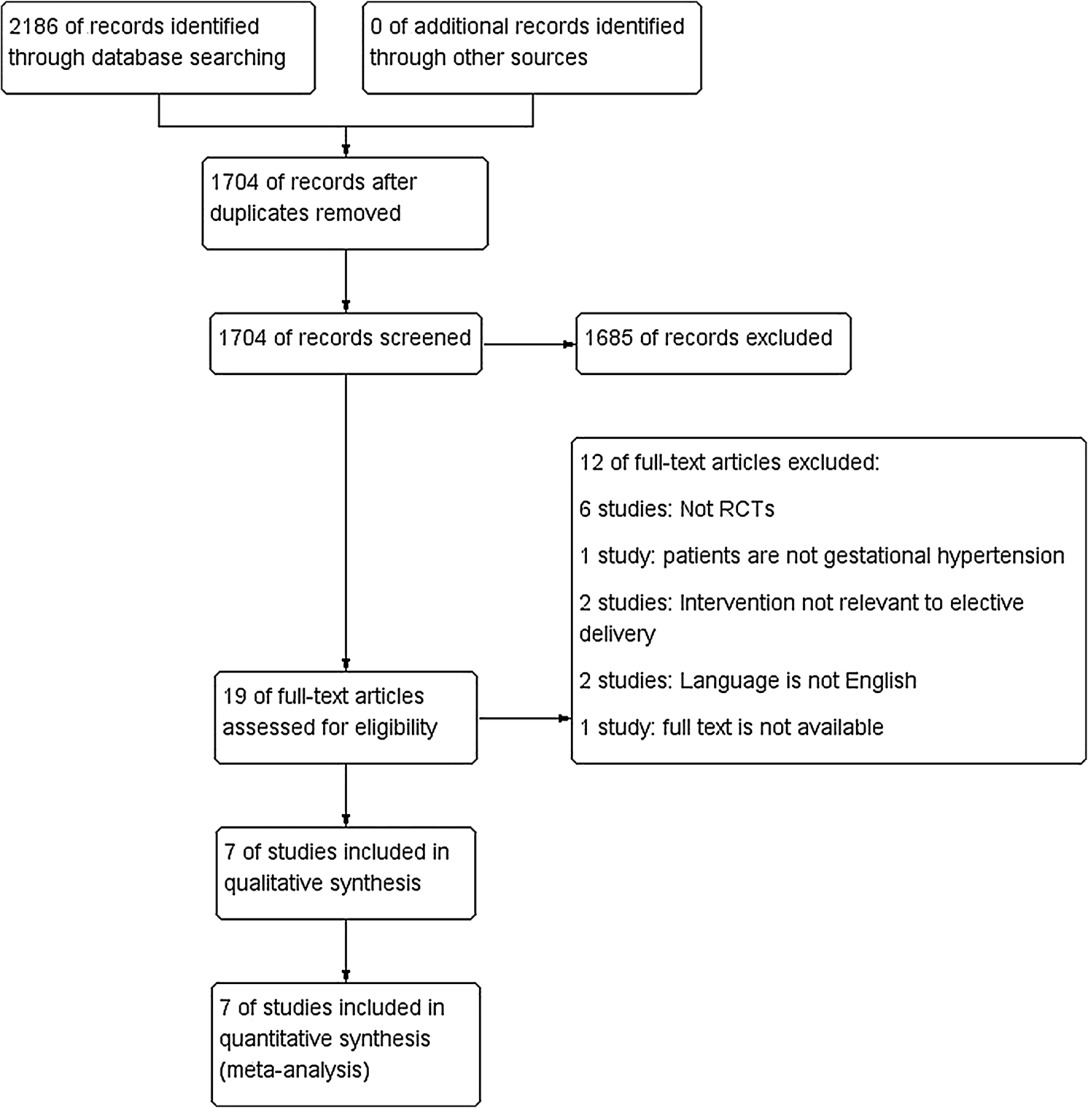
PRISMA flow diagram. After screening of duplicates and eligibility, seven studies were included in our analysis

The first phrase search yielded 1529 references after duplicates were removed and the updated search yielded 175 references of which seven studies were included in the final report (Fig. 1). The seven studies fulfilling our inclusion criteria were (HYPITAT-I 2009, Mesbah 2003, MEXPRE 2013, Odendaal 1990, Sibai 1994, Duvekot 2015 and a subset of participants from the GRIT study [18–27]). A total of 1,501 participants were included (range 30–756), 481 were diagnosed with severe PE, 264 with pre-eclampsia and severe hypertensive disorders, and 756 with PE and gestational hypertension (Table 3). One identified studies is on-going trial [28]. Therefore, six studies provided data [21–25, 27].

**Table 3:** Study characteristics table

Study ID (setting)	Participants		Definitions	Interventions		Other medications
	Length of pregnancy	Diagnosis *N* =	Pre-eclampsia or gestational hypertension	Interventionist *n*=	Expectant *n*=	
HYPITAT-I 2009 [18–20] (Netherlands)	36–41 weeks	Pre-eclampsia or gestational hypertension *N* = 756 Maternal age: median 29 (26–33) years Gestational age: median 38 (37–39) weeks	Pre-eclampsia: diastolic BP > 90 mm on two occasions at least 6 h apart; proteinuria (two or more occurrences of protein on a dipstick, >300 mg total protein within a 24-h urine collection, or ratio of protein to creatinine >30 mg/mmol) Gestational hypertension: diastolic BP ≧ 95 mmHg, on two occasions at least 6 h apart	Delivery by induction of labour within 24 h after randomisation. Labour induced by amniotomy with a Bishop cervix score >6 at vaginal examination *n* = 377	Monitored until the onset of spontaneous delivery; monitoring of blood pressure, screening of urine for protein with dipstick specimen or with ratio of protein to creatinine (inpatient or outpatient) *n* = 379	Use of oxytocin orprostaglandins depended on local protocols
Mesbah 2003 [21] (Egypt)	28–33 weeks	Severe pre-eclampsia *N* = 30 Maternal age: mean 24.7 ± 5.9 years Gestational age: mean 31.1 ± 1.7 weeks	Severe pre-eclampsia: BP > 180/120 mmHg on two occasions 30 min apart; or BP between 160 to 180/110 to 120 mmHg on two occasions six hours apart; > 500 mg of proteinuria on a 24 h urine collection measure	Administered dexamethasone phosphate; 48 h to lapse before either an induction of labour was attempted (50 µ, vaginal misoprostol) or caesarean section after 24 h *n* = 15	Administered dexamethasone phosphate then managed conservatively with bed rest, observations and nifedipine to control BP. Indications for delivery were imminent eclampsia, deteriorating renal function, spontaneous preterm labour, absent EDF or a non-reassuring CTG reaching 34 weeks *n* = 15	Blood pressure controlled with oral nifedipine
MEXPRE 2013 [22] (Latin America)*	28–33 weeks	Pre-eclampsia and severe hypertensive disorders N = 264 Maternal age: mean 28.15 ± 6.6 years Gestational age: mean 30.8 ± 1.6	Severe pre-eclampsia: BP > 140/90 mmHg or greater on two occasions at least 4 h apart and proteinuria >300 mg in 24 h urine specimen with 1 or more of the following additional criteria: BP > 160 mm Hg systolic or >110 mm Hg diastolic; proteinuria >5 g per 24 h; or symptoms suggesting significant end-organ involvement, such as headache, visual disturbances, epigastric pain, or tinnitus	‘Prompt delivery’: glucocorticoid therapy followed by delivery in 24–72 h, magnesium sulphate continued until 24 h after delivery *n* = 133 (*n* = 106 with severe pre-eclampsia)	Treated expectantly: glucocorticoid therapy followed by delivery only for specific maternal/ fetal indications or reaching 34 weeks of gestation *n* = 134 (*n* = 101 with severe pre-eclampsia)	Corticosteroids (betamethasone or dexamethasone Oral antihypertensive used in some centers (a-methyl dopa, nifedipine, or hidralazin)
Odendaal 1990 [23] (Africa)	28–34 weeks	Severe pre-eclampsia *N* = 38 Maternal age: mean 23 ± 4 years Gestational age: range 28–34 weeks	Severe pre-eclampsia: BP exceeding 180/120 mmHg on two occasions at least 30 min apart with 2 + or more proteinuria on dipstick Blood pressure of 160/110–180/120 mmHg on two occasions at least 6 h apart with 2 + or more proteinuria 150/100–160/110 mmHg on two occasions at least 6 h apart with 3 + or more proteinuria 140/90 mmHg or more proteinuria and clinical signs of imminent eclampsia (diagnosed in women with epigastric pain, severe headache, visual disturbances, nausea, and brisk tendon reflexes)	Delivery by induction or caesarean section depending on obstetric circumstances 48 h after betamethasone. If cervix not favourable, prostaglandin E_2_ tablets. If still not favourable after 24 h, caesarean section *n* = 20	Bed rest on high-risk obstetric ward; maternal and fetal condition monitored intensively; BP controlled with prazosin; delivery at 34 weeks unless indicated earlier *n* = 18	Magnesium sulphate (4 mg IV; 10 mg IM); dihydralazine (6.25 mg IV); betamethasone (12 mg IM)
Sibai 1994 [24] (USA)	28–32 weeks	Severe pre-eclampsia *N* = 95 Maternal age: mean 22.5 ± 5.1 years Gestational age: mean 30.1 ± 1.6 weeks	Severe pre-eclampsia: persistent elevations of blood pressure (systolic >160 mm Hg or diastolic -->- 110 mm Hg) during the initial 24 h of hospitalization. All had proteinuria (>500 mg per 24 h) and elevated serum uric acid levels (>5 mg/dl)	Delivery by caesarean section or by induction of labour, on the basis of obstetric condition, 48 h after first dose of betamethasone *n* = 46	Maternal and fetal monitoring on an antenatal ward. If either condition deteriorated, or reached 34 weeks’ gestation, delivery using the ‘most appropriate method’ *n* = 49	Betamethasone (12 mg at 24 h apart); labetalol (200 mg every 8 h, max. 2400 mg/day); nifedipine (max. 120 mg/day)
Duvekot 2015 [25]	28–32 weeks	Severe pre-eclampsia Maternal age: not reported Gestational age: 30 weeks	Severe pre-eclampsia: Not reported, as only abstract is available	Delivery 48 h after admission *n* = 26	Expectant management, no more details *n* = 30	Not reported
GRIT 2003 (Europe) [27 ]	≤34 weeks	Fetal compromise between 24 and 36 weeks *N* = 548 (*n* = 262 relevant with severe pre-eclampsia) Maternal age: median 28 (24–32) years Gestational age: median 32 (29–34) weeks	Not reported—gestational age between 24 and 36 weeks, umbilical artery Doppler waveform recorded	Delivery within 48 h to permit completion of a steroid course *n* = 141	Delayed delivery until obstetrician no longer uncertain *n* = 121	Not reported

MEXPRE 2013*—additional definitions: Severe gestational hypertension defined as: BP > 140/90 mmHg or greater on two occasions at least 4 h apart and <300 mg of protein in a 24 h urine specimen with 1 or more of the following additional criteria: BP > 160 mmHg systolic greater than 110 mm Hg diastolic; or symptoms suggesting significant end-organ involvement. Chronic hypertension: hypertension present before pregnancy/ before the 20th week of gestation. Superimposed pre-eclampsia in women with chronic hypertension: development of new-onset proteinuria, with 1 or more of the following criteria: blood pressure greater than 160 mm Hg systolic or greater than 110 mm Hg diastolic; or symptoms suggesting significant end-organ involvement

### Study characteristics

In the six studies that provided data for women with severe PE who were preterm delivery, definitions of the disorder were comparable between studies. Maternal and gestational ages as well as the length of pregnancies were also fairly uniform (Table 3) [21–24, 27]. One study provided data for women with PE who were pre- and post-term, and gestational hypertension between 36 and 41 weeks [18–20, 26]. Procedures for elective delivery in the intervention groups with severe pre-eclampsia were comparable among the five studies, with delivery required within 24–72 h after the admission or after administration of steroids. Elective delivery was either via induction of labour or caesarean section (CS). The choice to induce labour or perform a CS was based on obstetrical indications, such as malpresentation, hypertensive disorders, and dystocia [21–24]. In one study of women with pre-eclampsia, labour was induced within 24 h following randomization [18–20, 26].

### Maternal death and eclampsia

There were no maternal deaths or strokes in any of the included studies in women with severe PE, PE or gestational hypertension (*n* = 1020). Three studies reported the incidence of eclampsia; only one study reported one case of eclampsia in each group of MEXPRE 2013 in women less than 34 weeks gestation with severe PE [21], however, the difference was not significant (*n* = 389, 3 RCTs, RR 1.02, 95 CI 0.06–16.06, *p* = 0.99).

### Maternal complications

**Fig. 2:**
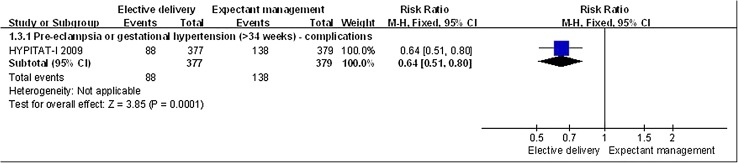
Elective delivery versus expectant management, maternal outcome: PE related complications. PE related complications were decreased in puerperas that elective delivery compared to in puerperas that expectant management (RR < 1)

In pregnant women with PE greater than 34 weeks gestation, elective delivery significantly lowered the incidence of any maternal complication (*n* = 756, 1 RCT; RR, 0.64; 95% CI 0.51–0.80, *p* = 0.0001; Fig. 2). Though elective delivery was also associated with a lower incidence of HELLP syndrome in women with PE or gestational hypertension greater than 34 weeks gestation (1.06 vs 2.9%), the difference was not significant (*n* = 756, 1 RCT; RR,0.37; 95% CI 0.12–1.14, *p* = 0.08).

**Fig. 3:**
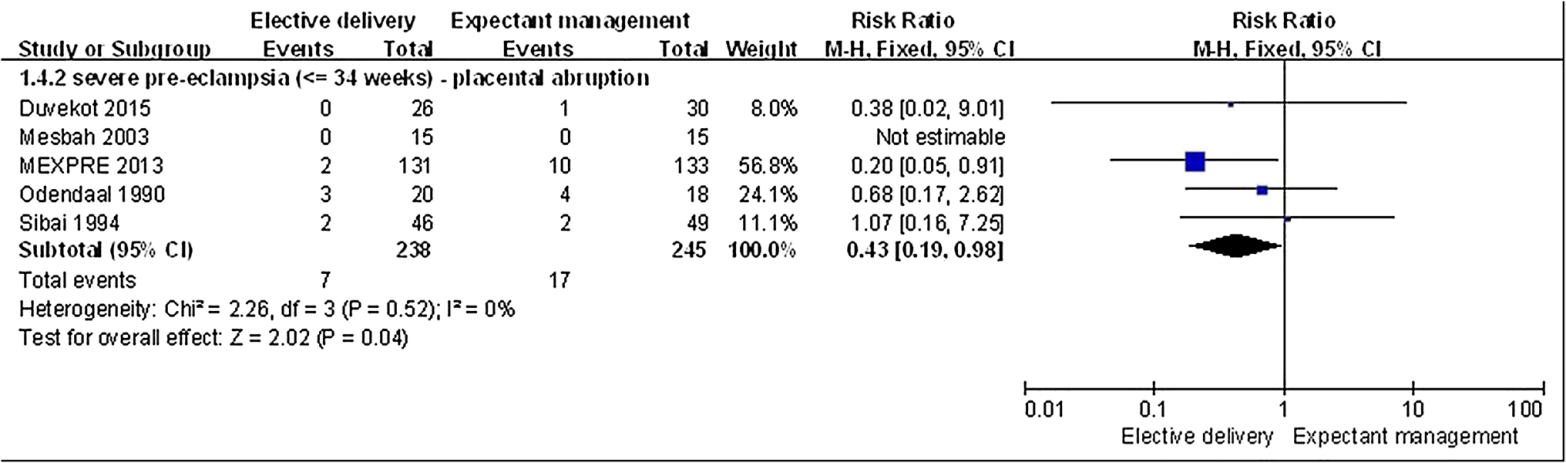
Elective delivery versus expectant management, maternal outcome: placental abruption. Occurrence of placental abruption in puerperas that elective delivery was lower than in puerperas that expectant management (RR < 1)

In women with severe PE less than 34 weeks gestation, elective delivery was associated with a significantly lower incidence of placental abruption (*n* = 483, 5 RCTs; RR, 0.43; 95% CI 0.19–0.98, *p* = 0.04; Fig. 3). There was no significant difference in the incidence of renal failure (*n* = 427, 4 RCTs; RR, 0.33; 95% CI 0.05–2.03, *p* = 0.23) or HELLP syndrome (*n* = 389, 3 RCTs; RR, 1.12; 95% CI 0.64–1.97, *p* = 0.69) between the management groups. There was no incidence of disseminated coagulopathy in the elective delivery group. Two patients in the expectant management group experienced this complication, however, this difference was not significant (*n* = 359, 2 RCTs; RR, 0.20; 95% CI 0.01–4.17, *p* = 0.30).

There were no significant differences between the management groups in the incidence of pulmonary edema in women with severe PE less than 34 weeks gestation (*n* = 415, 3 RCTs; RR, 0.46; 95% CI 0.07–3.05, *p* = 0.42) or PE / hypertension greater than 34 weeks gestation (*n* = 756, 1 RCT; RR, 0.20; 95% CI 0.01–4.17, *p* = 0.30). The incidence of postpartum hemorrhage (>500 ml blood loss) was also similar between the groups in women with PE or hypertension greater than 34 weeks (*n* = 756, 1RCT; RR, 0.88; 95% CI 0.57–1.35, *p* = 0.56).

### Maternal hypertension

Women with PE or gestational hypertension greater than 34 weeks who underwent elective delivery experienced less of an increase in both diastolic (*n* = 756, 1 RCT; RR, 0.61; 95% CI 0.46–0.80, *p* = 0.0005) and systolic blood pressure (*n* = 756, 1 RCT; RR, 0.63; 95% CI 0.46–0.85, *p* = 0.003). There was also a significant difference between the management groups in terms of a requirement for antihypertensive drug therapy. Significantly fewer women greater than 34 weeks gestation with PE or hypertension who underwent elective delivery required either oral or intravenous antihypertensive drug therapy (*n* = 756, 1 RCT; RR, 0.61; 95% CI 0.46–0.79, *p* = 0.0003) and (*n* = 756, 1 RCT; RR, 0.34; 95% CI 0.18–0.62, *p* = 0.0005), respectively. This was also the case for women less than 34 weeks gestation with severe PE (*n* = 264, 1 RCT; RR, 0.01; 95% CI 0.00–0.13, *p* = 0.0006).

### Fetal and neonatal mortality

The pooled analysis indicated that there was no any difference in the incidence of fetal or neonatal mortality between the management groups in women less than 34 weeks gestation with severe pre-eclampsia (*n* = 689, 5 RCTs; RR, 0.30; 95% CI 0.07–1.22, *p* = 0.09) and (*n* = 485, 5 RCTs; RR, 1.34; 95% CI 0.82–2.20, *p* = 0.24), respectively. There were no neonatal or fetal deaths reported in women greater 34 weeks’ gestation with PE or gestational hypertension (*n* = 756, 1 RCT).

### Neonatal complications

**Fig. 4:**
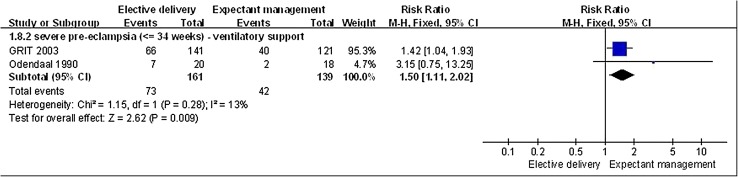
Elective delivery versus expectant management, neonatal outcome: ventilated. Occurrence of ventilated in neonates that undergo elective delivery was higher than in neonates that undergo expectant management (RR > 1)

Overall estimates showed that there was no difference between the management groups in the incidence of neonatal necrotising enterocolitis in women less than 34 weeks gestation with severe PE (*n* = 659, 4 RCTs; RR, 1.78; 95% CI 0.83–3.79, *p* = 0.14). However, neonates whose mother’s underwent elective delivery for severe PE at less than 34 weeks gestation required more ventilatory support than neonates whose mother’s in this group were managed expectantly (*n* = 300, 2 RCTs; RR, 1.50; 95% CI 1.11–2.02, *p* = 0.009; Fig. 4). There was no significant difference in the incidence of cerebral hemorrhage (*n* = 95, 1 RCT; RR, 3.20; 95% CI 0.34–29.63, *p* = 0.31) between the management groups in neonates of mother’s less than 34 weeks gestation with severe PE.

**Fig. 5:**
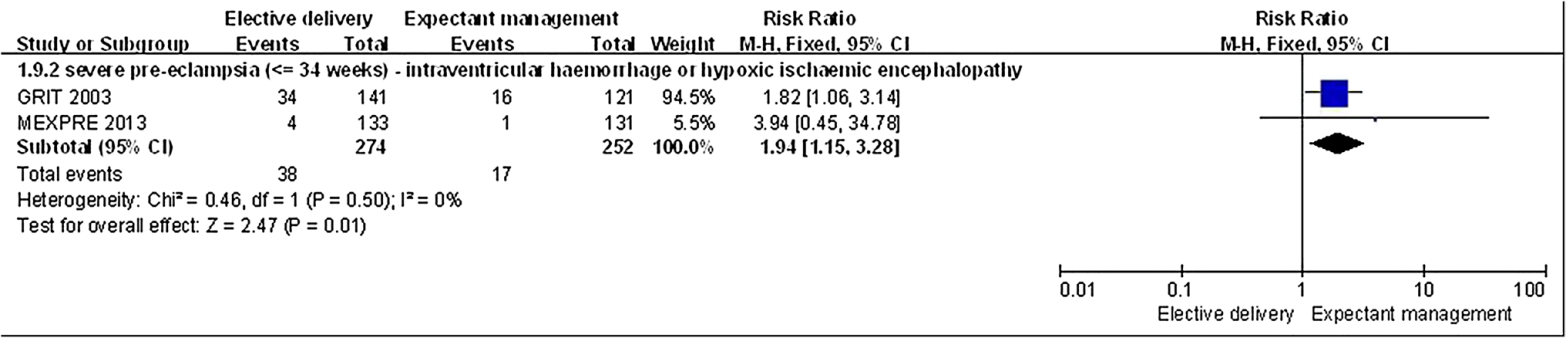
Elective delivery versus expectant management, neonatal outcome: interventricular hemorrhage or hypoxic ischemic encephalopathy. Interventricular hemorrhage or hypoxic ischemic encephalopathy in neonates that undergo elective delivery was higher than in neonates that undergo expectant management (RR > 1)

Neonates whose mother’s underwent an elective delivery at less than 34 weeks for severe PE had a higher incidence of interventricular hemorrhage or hypoxic ischemic encephalopathy compared to neonates whose mothers in this group were managed expectantly (*n* = 526, 2 RCTs; RR, 1.94; 95% CI 1.15–3.28, *p* = 0.01; Fig. 5).

In neonates whose mothers were admitted with severe PE less than 34 weeks gestation, the incidence of hyaline membrane disease was similar in both management groups (*n* = 397, 3 RCTs, RR 1.66, 95% CI 0.92–2.99, *p* = 0.09). There was also no significant difference between the management groups in the incidence of bronchopulmonary dysplasia (*n* = 95, 1 RCT; RR, 2.13; 95% CI 0.41–11.08, *p* = 0.37) or pneumothorax (*n* = 40, 1 RCT; RR, 3.00; 95% CI 0.34–26.45, *p* = 0.32) in this group of women. Significantly more neonates whose mothers were managed expectantly with severe PE at less than 34 weeks gestation were small-for-gestational age (SGA) (*n* = 389, 3 RCTs; RR, 0.37; 95% CI 0.23–0.60, *p* < 0.0001).

### Risk of bias assessment

**Fig. 6:**
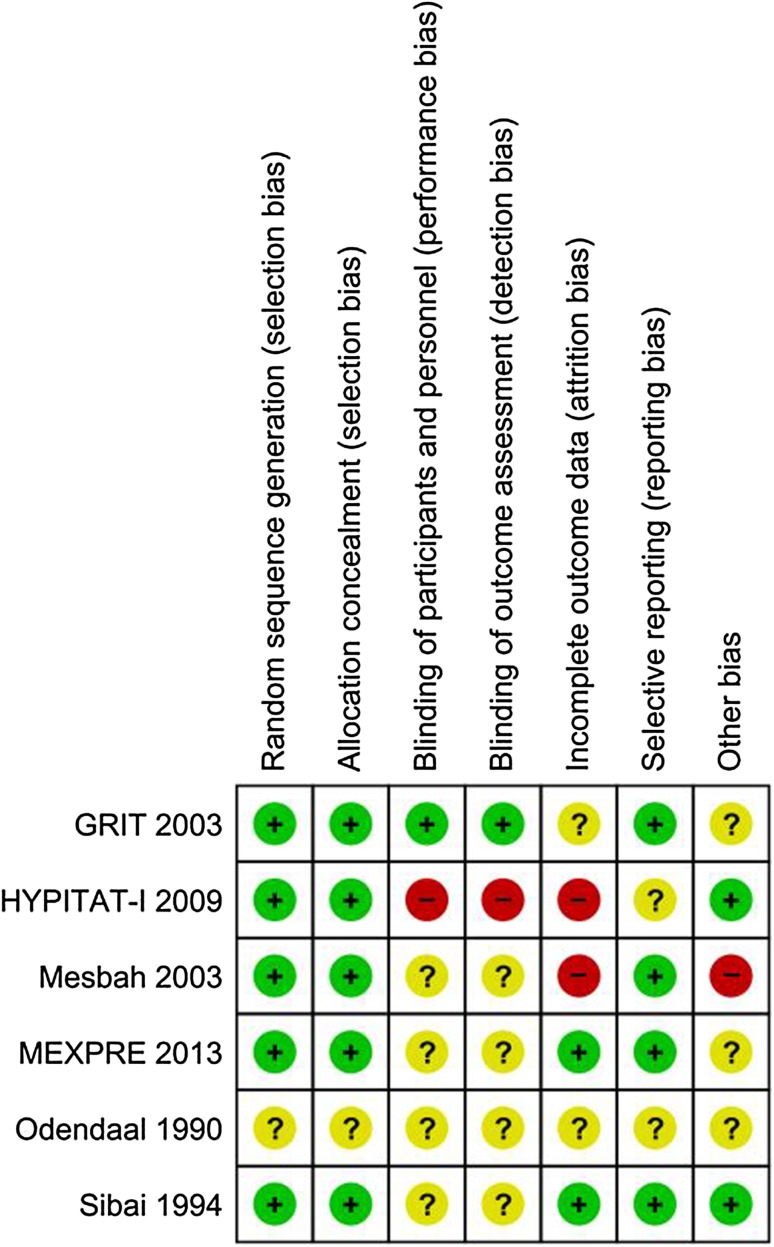
Risk of bias assessment: provides a graphical overview of the risk of bias rating within each included study

Six out of the seven studies adequately described the method of randomization, and two studies were rated as a ‘high’ risk of bias across one or more of the pre-specified domains examining methodological quality (Fig. 6 provides a graphical overview of the risk of bias rating within each included study). One study expressly mentioned an ‘open-label’ design with regards to blinding participants [18–20, 26], however, all of the remaining studies failed to mention the blinding methods used. Due to the nature of the intervention, it is clear that any level of double blinding is not possible or indeed ethical. However, one study did mention that an attempt was made to blind the treatment allocator to the data abstracter and neonatologist [22]. One study selectively excluded women with a more severe level of PE, after elective delivery via CS [21]. Risk of bias assessment was not possible with all included studies due to a lack of methodological detail in the studies’ design. These are all factors that may increase the risk of bias in included studies and affect the quality of the evidence.

### Quality assessment of the evidence

We have summarized the quality of evidence for the primary maternal outcomes in Table 4.

**Table 4:** Summary of findings table

Quality assessment							No of patients		Effect		Quality	Importance
No of studies	Design	Risk of bias	Inconsistency	Indirectness	Imprecision	Other considerations	Interventionist care versus expectant care	Control	Relative (95% CI)	Absolute		
Maternal: eclampsia—severe pre-eclampsia (≦34 weeks)												
3	Randomised trials	No serious risk of bias	No serious inconsistency^a^	No serious indirectness	Serious^b^	None	1/192 (0.52%)	1/197 (0.51%)	RR 1.02 (0.06 to 16.06)	0 More per 1000 (from 5 fewer to 76 more)	⊕⊕⊕O Moderate	Critical
								0%		−		
Maternal: complications or progression to severe disease—pre-eclampsia or gestational hypertension (>34 weeks)												
1	Randomised trials	Serious^c^	No serious inconsistency	No serious indirectness	No serious imprecision	None	88/377 (23.3%)	138/379 (36.4%)	RR 0.64 (0.51 to 0.8)	131 Fewer per 1000 (from 73 fewer to 178 fewer)	⊕⊕⊕O Moderate	Critical
								36.4%		131 Fewer per 1000 (from 73 fewer to 178 fewer)		
Maternal: placental abruption—severe pre-eclampsia (≦34 weeks)												
4	Randomised trials	No serious risk of bias	No serious inconsistency	No serious indirectness	No serious imprecision	None	7/212 (3.3%)	16/215 (7.4%)	See comment	43 Fewer per 1000 (from 90 fewer to 0 more)	⊕⊕⊕⊕ High	Critical
								5.8%		34 Fewer per 1000 (from 70 fewer to 0 more)		
Maternal: renal failure—severe pre-eclampsia (≦34 weeks)												
4	Randomised trials	No serious risk of bias	No serious inconsistency	No serious indirectness	Serious^d^	None	1/212 (0.47%)	4/215 (1.9%)	RR 0.33 (0.05 to 2.03)	12 Fewer per 1000 (from 18 fewer to 19 more)	⊕⊕⊕O Moderate	Critical
								1.1%		7 Fewer per 1000 (from 10 fewer to 11 more)		
Maternal: HELLP Syndrome—severe pre-eclampsia (≦34 weeks)												
3	Randomised trials	No serious risk of bias	No serious inconsistency	No serious indirectness	Serious^e^	None	22/192 (11.5%)	20/197 (10.2%)	RR 1.12 (0.64 to 1.97)	12 More per 1000 (from 37 fewer to 98 more)	⊕⊕⊕O Moderate	Critical
								4.1%		5 More per 1000 (from 15 fewer to 40 more)		
Maternal: HELLP Syndrome—pre-eclampsia or gestational hypertension (>34 weeks)												
1	Randomised trials	Serious^f^	No serious inconsistency	No serious indirectness	Serious^g^	None	4/377 (1.1%)	11/379 (2.9%)	RR 0.37 (0.12 to 1.14)	18 Fewer per 1000 (from 26 fewer to 4 more)	⊕⊕OO Low	Critical
								2.9%		18 Fewer per 1000 (from 26 fewer to 4 more)		
Maternal: disseminated coagulopathy—severe pre-eclampsia (≦34 weeks)												
2	Randomised trials	No serious risk of bias	No serious inconsistency	No serious indirectness	Serious^h^	None	0/177 (0%)	2/182 (1.1%)	RR 0.2 (0.01 to 4.19)	9 Fewer per 1000 (from 11 fewer to 35 more)	⊕⊕⊕O Moderate	Important
								0.8%		6 Fewer per 1000 (from 8 fewer to 26 more)		
Maternal: pulmonary oedema—severe pre-eclampsia (≦34 weeks)												
2	Randomised trials	No serious risk of bias	No serious inconsistency	No serious indirectness	Serious^i^	None	1/177 (0.56 %)	2/182 (1.1%)	RR 0.51 (0.05 to 5.53)	5 Fewer per 1000 (from 10 fewer to 50 more)	⊕⊕⊕O Moderate	Important
								0.8%		4 Fewer per 1000 (from 8 fewer to 36 more)		
Maternal: postpartum hemorrhage (≥500 ml blood loss)—pre-eclampsia or gestational hypertension (>34 weeks)												
1	Randomised trials	Serious^f^	No serious inconsistency	No serious indirectness	No serious imprecision	None	35/377 (9.3%)	40/379 (10.6%)	RR 0.88 (0.57 to 1.35)	13 Fewer per 1000 (from 45 Fewer to 37 more)	⊕⊕⊕O Moderate	Critical
								10.6%		13 Fewer per 1000 (from 46 fewer to 37 more)		

^a^Inconsistency: downgraded one level due to only one study providing data

^b^Imprecision: downgraded one level due to small sample size

^c^Risk of bias: downgraded one level due to high risk of bias in blindness of patients, personnel and outcome assessor

^d^Imprecision: downgraded one level due to small sample size

^e^Imprecision: downgraded one level due to small sample size

^f^Risk of bias: downgraded one level due to only one study providing data

^g^Imprecision: downgraded one level due to small sample size

^h^Imprecision: downgraded one level due to small sample size

^i^Imprecision: downgraded one level due to small sample size

## Discussion

This meta-analysis evaluated the effectiveness of elective delivery versus expectant management performed either before or after 34 weeks of gestation in women with PE in general or severe PE. Below we summarize our findings by patient outcomes. With respect to maternal outcomes, the incidence of eclampsia was similar for elective delivery and expectant management across all patient groups. The evidence for this finding is graded as moderate. For the prevention of maternal complications (e.g. placental abruption, HELLP syndrome), moderate evidence has suggested that elective delivery significantly reduced the incidence of all maternal complications compared with expectant management after 34 weeks of gestation in women with PE in general; high quality evidence has suggested that elective delivery also significantly decreased the incidence of placental abruption before 34 weeks of gestation in women with severe PE. No significant differences between the two interventions were found in preventing other maternal complications (renal failure, HELLP syndrome, disseminated coagulopathy, pulmonary edema and postpartum hemorrhage) regardless of gestation age or PE severity. This result was supported by moderate or low quality evidence. Regarding the management of hypertension, elective delivery was associated with significantly less increase in diastolic and systolic blood pressure and lower rates of antihypertensive drug therapy than expectant delivery after 34 weeks of gestation in women with PE; elective delivery was also associated with significantly fewer patients requiring antihypertensive drug therapy before 34 weeks of gestation in women with severe PE. These results were supported by moderate evidence.

With respect to neonatal outcomes, evidence was only available for before 34 weeks of gestation in women with severe PE. No difference existed between elective delivery and expectant management in fetal and neonatal mortality. For the prevention of neonatal complications, elective delivery resulted in significantly higher rate of ventilation use and interventricular hemorrhage/hypoxic ischemic encephalopathy than expectant management before 34 weeks of gestation in women with severe PE. However, expectant management was associated with a significantly increased incidence of small neonates for their gestation age compared with elective delivery in the same patient population. No significant differences between the two interventions were found in preventing other neonatal complications, including necrotizing enterocolitis, cerebral hemorrhage, hyaline membrane disease, bronchopulmonary dysplasia, or pneumothorax before 34 weeks of gestation in women with severe PE.

One Cochrane review, published in 2013, compared the outcomes of elective delivery versus expectant management in women with severe PE between 24 and 34 weeks’ gestation [14]. This review included four small trials with a total of 425 patients. Because of the small sample size, the evidence was insufficient to permit reliable conclusions on maternal outcomes. Regarding neonatal outcomes, the Cochrane review reported that in women with severe PE between 24 and 34 weeks of gestation, elective delivery was associated with increased neonatal morbidity relative to expectant management. In comparison to the Cochrane review, this analysis included two additional recent, large RCTs (*n* = 756, and *n* = 264) in the addition of 1020 patients [18, 22]. This allows us to assess both maternal and neonatal outcomes. Our assessment of neonatal outcomes indicated that elective delivery resulted in a higher rate of neonatal complications than expectant management before 34 weeks’ gestation in women with severe PE, which is consistent with the Cochrane review. However, our analysis also revealed that elective delivery may decrease the incidence of placental abruption in the same patient population. In light of the dilemma in maternal and neonatal outcomes, clinicians should carefully balance the risks versus benefits of elective delivery in women with severe PE before 34-weeks’ gestation to achieve optimal outcomes for both the mother and baby. Given that the risk of placental abruption may outweigh that of neonatal complications, elective delivery could be more beneficial than expectant management to high risk women with severe PE before 34 weeks’ gestation.

An additional strength of our review is that we used the GRADE system to rate the quality of the evidence base. When providing guidance for clinical decision making, a recommendation should inform the clinicians of not only the benefits and risks associated with a particular intervention but also the reliability of that recommendation. Failure to consider the quality of the evidence base on which the recommendation is derived may lead to misguidance [29]. Our rating of the evidence base using the GRADE system has provided the clinicians with a concise summary of the quality of the evidence without including unnecessary details.

Some changes were made to the protocol of this review after registration [11]. To improve clarity and distinguish this review from the existing Cochrane review, we changed the title from ‘interventionist versus expectant management for pre-eclampsia’ to ‘elective delivery versus expectant management for pre-eclampsia.

The limitations of this analysis should be mentioned. In our search strategy, we specifically searched for studies including participants with a diagnosis of PE. Therefore, we may have overlooked some studies that included an unspecified subset of women with PE. Another limitation is that our general PE group included patients with gestational hypertension. Unlike the Cochrane review, our analysis intended to compare elective delivery and expectant management in women with PE, in general, in addition to women with severe PE. One large RCT in our search results, the HYPITAT-I trial allowed for this comparison [18]. However, the enrollment criterion for the HYPITAT-I trial was gestational hypertension or mild PE. Because this trial did not report separate outcomes for the two conditions, it is impossible to tease out the outcomes for PE only. Also, the women included in this study had a gestation age between 36 and 41 weeks, which was slightly above the median gestational age in other studies that contributed data to the patient group above 34 weeks? Gestation [18]. Given the consideration that studies including women with non-severe PE are sparse, and that this study has directly informed national and international guidelines on recommendations for PE management, we decided to include this study in our analysis despite its mixed patient population (PE and gestational hypertension). Including the HYPITAT-I trial offered the benefit of adding 756 patients; this large sample size permitted a conclusion in women with PE in general. Moderate evidence demonstrated that elective delivery may prevent maternal complications in women with PE beyond 34 weeks of pregnancy. However, it should be noted that the general PE group in our analysis included patients with gestational hypertension.

We are unable to report the outcomes in women with severe PE beyond 34 weeks of gestation because of the lack of evidence, although these outcomes are within the initial scope of this analysis. The absence of evidence partially ascribes to the termination of pregnancy typically performed in this patient population. As termination of pregnancy is recommended in women with severe PE beyond 34 weeks of gestation by the Federation of Gynecology and Obstetrics, expectant management is rarely used in this population. Since evidence suggests that elective delivery is generally more beneficial than expectant management in women with severe PE below 34 weeks of gestation, it is reasonable to speculate that elective delivery should also be recommended in severe PE patients beyond 34 weeks of gestation. In summary, our analysis provides insights into the effectiveness of elective delivery versus expectant management, as well as the timing of the interventions in managing PE, in general, and severe PE. However, future studies will be required to verify the results of our analysis. The studies that can provide strong evidence should be RCTs with a large sample size, adequate randomization, and outcome assessors blinded to the treatments. Although our analysis included studies of non-severe PE, the number of these studies is low. We identified only one appropriate study of non-severe PE in women before 34 weeks of gestation and one in women beyond that gestation age, and both studies included women with gestational hypertension [18, 27]. Therefore, a convincing conclusion on PE, in general, requires more RCTs conducted in a homogenous PE patient population (preclude gestational hypertension) but including patients with severe and non-severe PE. Regarding outcomes, more RCTs will be required to report on any maternal complications, increase in blood pressure and postpartum hemorrhage, particularly, in patients with severe PE.

## Conclusion

Elective delivery is generally more beneficial than expectant management for women with PE or gestational hypertension beyond 34 weeks of gestation and women with severe PE. This intervention can reduce the risk of PE-related complications and lower the incidence of severe hypertension and the need for antihypertensive medication in women with PE beyond 34 weeks of gestation; it can also reduce the risk of placental abruption in women with severe PE before 34 weeks of gestation. However, elective delivery may increase the rate of ventilation use and the risk of interventricular hemorrhage/hypoxic ischemic encephalopathy in neonates. More data from RCTs with larger sample sizes will be required to further evaluate the benefits and harm of elective delivery versus expectant management for women and neonatal outcomes.
